# *Plasmodium falciparum* remains the dominant parasite affecting children despite decades of implementing vector control in two villages of Wolaita Zone, Southwest Ethiopia

**DOI:** 10.3389/fepid.2023.1305074

**Published:** 2024-01-05

**Authors:** Shimels Elias, Fekadu Massebo

**Affiliations:** Department of Biology, Arba Minch University, Arba Minch, Ethiopia

**Keywords:** Humbo district, malaria prevalence, IRS, *Plasmodium falciprum*, Wolaita Zone

## Abstract

**Background:**

Malaria is still a significant public health concern, and its prevention and control measures have different impacts in different areas. This study assesses the prevalence of malaria and the effectiveness of routine malaria control programmes such as indoor residual spray (IRS) in two Ethiopian villages.

**Methods:**

The *Kebeles* (villages) were purposefully selected based on their malaria prevalence rates. A parasitology survey was conducted in Fango-Gelchecha pre- and post-IRS implementation, whereas in Shochora-Abela it was only conducted post-IRS implementation. The IRS was implemented as part of the routine malaria control programme in August 2017. Every fourth house from the village registration list was systematically selected, resulting in a sample of 300 households per village. A total of 3,075 individuals were enrolled for malaria testing using microscopy.

**Results:**

After three to four months of application of IRS in August 2017, 59 malaria cases were confirmed, resulting in an overall prevalence of 1.9% (95% CI: 1.5–2.5). Of the positive cases, 18 cases (0.59%: 95% CI: 1.3–1.8) were from Shochora-Abela village, and 41 cases (1.33%: 95% CI: 1.1–1.3) were from Fango-Gelchecha. About age categories, the prevalence of malaria was 10.1% (95% CI: 5.9–15.9) among children under five, 4.7% (95% CI: 3.3–6.4) in children aged 5–14, and only 0.32% (95% CI: 0.13–0.67) in the age group 15 and above. Overall, *P. falciparum* was the dominant malaria parasite, accounting for 69.5% (95% CI: 56.1–80.8), while *P. vivax* malaria accounted for 30.5% (95% CI: 19.2–43.8). The malaria prevalence in Fango-Gelchecha village was 3.1% (95% CI: 2.3–4.0) before IRS and 2.6% (95% CI: 1.8–3.5) after IRS application. In the village of Shochora-Abela, the prevalence of malaria post-IRS was 1.2% (95% CI: 0.7–1.9), but the prevalence prior to IRS was not evaluated.

**Conclusions:**

*Plasmodium falciparum* is the predominant parasite in the villages, mainly affecting children under five. Therefore, protecting young children should be the top priority for reducing infection burdens.

## Introduction

Malaria is a prevalent vector-borne disease causing nearly half a million deaths annually, with Africa accounting for over 90% of all deaths (1, 2). Although malaria deaths among children under five have decreased, this age group is still the most vulnerable to infection and fatality in high-transmission regions (2). Five species of *Plasmodium* can infect humans, with *P. falciparum* causing the majority of malaria deaths (1). In Ethiopia, the two most important parasites are *P. falciparum* and *P. vivax* (3). *Anopheles* mosquitoes transmit *Plasmodium* to humans, with *An. arabiensis* being the primary malaria vector (4–6) and *An. pharoensis* playing a minor role (7).

Vector control interventions have significantly impacted malaria epidemiology (8), but their effectiveness varies based on vector behavior, intervention method usage, and human behavior (9). The proper use of tools reduce malaria, but drug-resistant parasites, insecticide-resistant vectors, and changes in mosquito behavior can limit success (10–12).

Although there have been efforts to decrease malaria-related sickness and deaths, it still remains a common cause for outpatient visits in southwest Ethiopia (13). Wolaita, located in southwest Ethiopia, has a high prevalence of malaria, with *P. falciparum* and *P. vivax* being the two most common parasite species. A ten-year trend analysis of malaria from health facilities showed that *P. falciparum* was the dominant parasite, accounting for 72%, while *P. vivax* accounted for 24%, and mixed infections of the two parasites accounted for 4% (14). For over 20 years, the region has extensively used indoor residual spraying (IRS) with different insecticides and long-lasting insecticide nets (LLINs) as intervention strategies. Since 2005, rapid diagnostic test (RDT) have been used for prompt diagnosis and treatment of patients with effective antimalarial drugs such as Artemisinin-based combination therapies (ACT) at community-based primary health care facilities (3). In the past, *P. falciparum* was the most common parasite responsible for causing malaria (15). However, due to intensive malaria interventions in some areas, there has been a shift in the dominant parasite species. Recently, there has been a consistent increase in cases of *P. vivax* malaria, which is caused by various factors, including the unique biology of *P. vivax* malaria (16). Although children under five and mothers are usually at significant risk of malaria infection, there has been a shift in the burden of cases (17). Therefore, to achieve the country's malaria control and elimination targets, it is important to understand the current dynamics of malaria parasite species and the population at highest risk of infections. Thus, this study aimed to assess the prevalence of malaria among all age groups, identify the proportion of *Plasmodium* species and the imapct of the IRS intervention implemented by routine malaria control system in reducing malaria prevalence in two malaria- endemic *Kebeles* (villages) located in the Humbo district of the Wolaita zone in southwest Ethiopia.

## Materials and methods

### Description of the study area

The study was conducted in the Humbo district of the Wolaita Zone in the Southern Nation Nationalities and People Regional state (SNNPRs). The district has an altitude ranging from 1,001 to 2,700 meters above sea level (masl). The district comprises two urban and 39 rural villages with an average annual minimum temperature of 15°C and maximum of 31°C. The yearly minimum rainfall averages was 14.3m^3^, while the maximum was 27.46m^3^.

**Figure 1:**
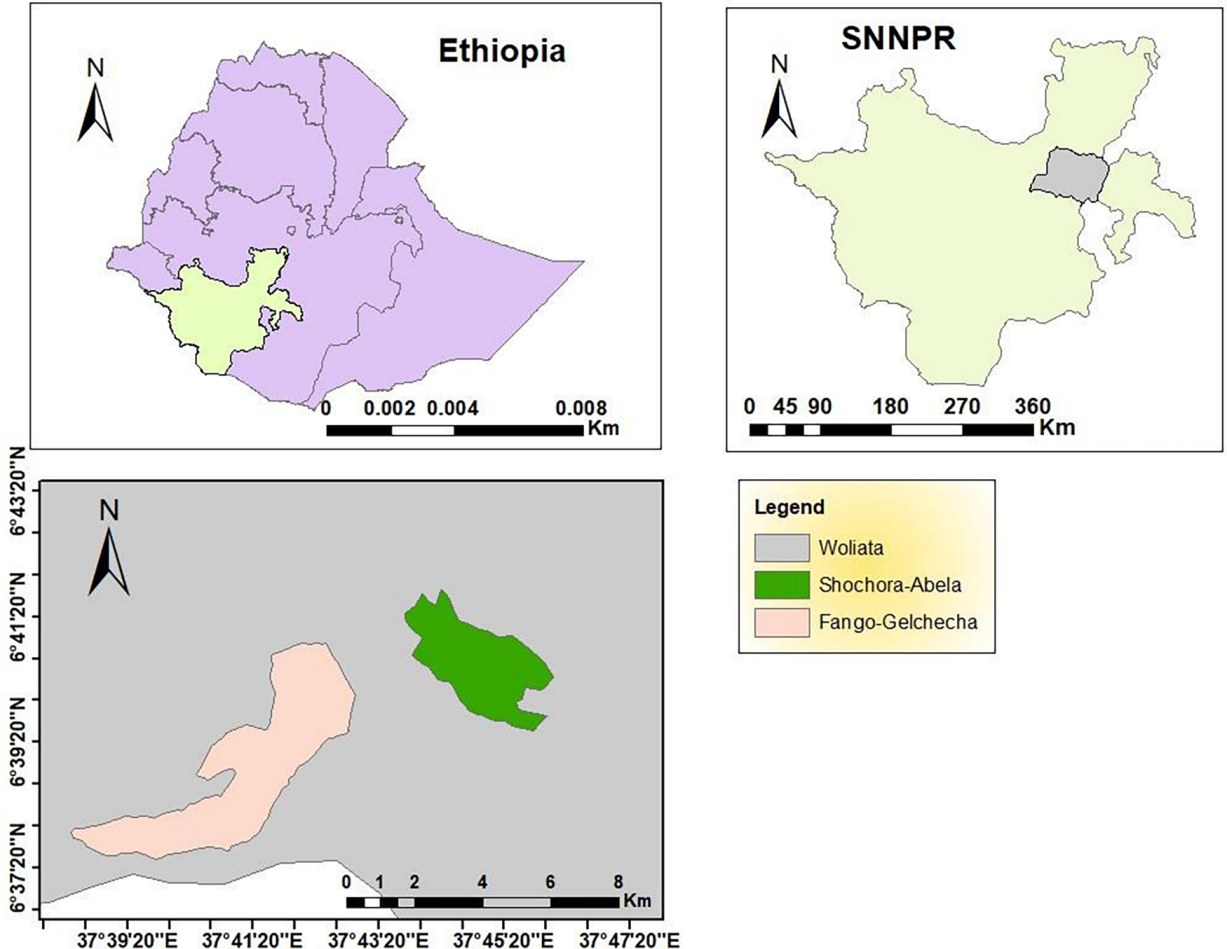
Map of the study villages in humbo district, wolaita zone in southwest Ethiopia.

The study includes two rural villages, Fango-Gelchecha and Shochora-Abela (Figure 1), with a total population of 11,075 and 10,975, respectively. Fango-Gelchecha is located at 06°35.565 longitude and 037°33.963 latitude, with a village centre elevation of 1,264 masl; while Shochora-Abela is located at 06°35.936 longitude and 037°46.665 latitude, with a village centre elevation of 1,456 masl. The primary occupations of the inhabitants are agriculture, trading, and cattle ranching.

Malaria is a prevalent disease in the two villages. The main methods of controlling the malaria vector are IRS and LLINs. Free malaria diagnosis and treatment are offered at each health post.

### Study design

**Figure 2:**
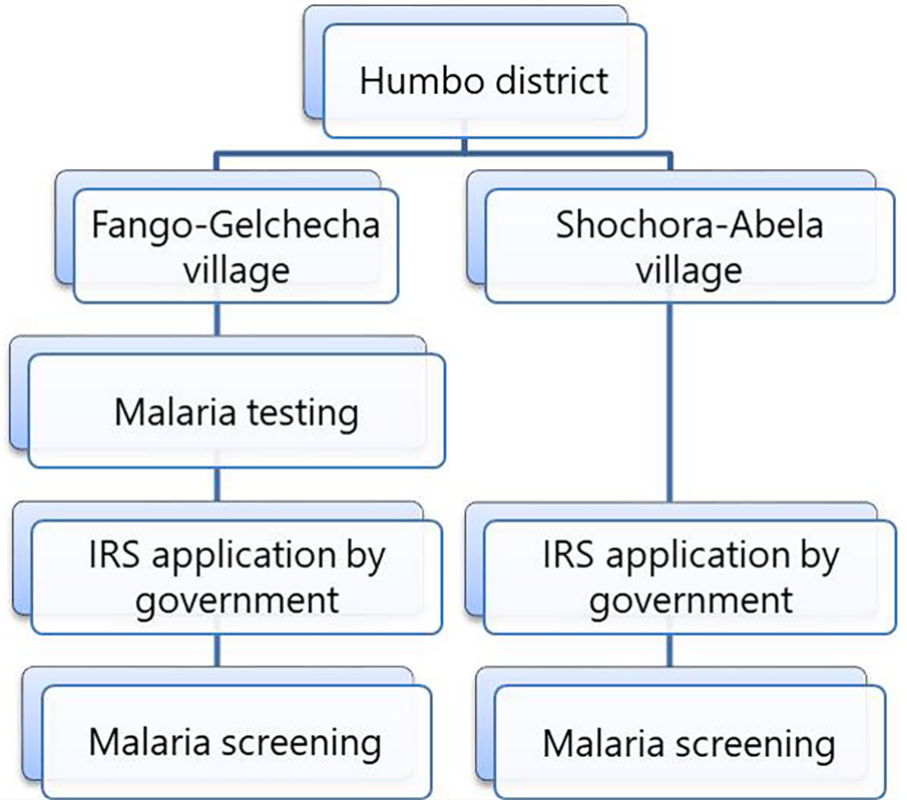
Study flow chart.

A survey was conducted from August to December 2017 to determine malaria prevalence in two Humbo villages. The villages were selected based on prevalence of malaria and households were selected using systematic random sampling with a village registration list as a sampling frame. Six hundred households from two villages were systematically selected for malaria parasite testing. The first household was picked from the registration list, and subsequent households were included by dividing the total number of households by the assigned household number in each village. The parasitological survey was conducted twice in Fango-Gelchecha village: once before IRS in August 2017 and again in November and December 2017, after the IRS. In Shochora-Abela village, the survey was done only after the IRS application (Figure 2) due to logistical constraints. The propoxur IRS was implemented in August 2017 as part of the routine malaria control programme. Although the routine malaria control programme conducted the IRS application, it is worth reporting the impact of the intervention.

Data on malaria prevalence was collected in both villages after the IRS application in November and December 2017. The data collected before the IRS in Fango-Gelchecha was used to assess its effect. Blood samples were collected through house-to-house visits. The questionnaire was designed to gather information about the participants' socio-demographic characteristics, bed net usage, and IRS. The questionnaire was initially developed in English and translated into Amharic.

### Sample size determination

The sample size for estimating population prevalence was calculated using Bartlett et al.'s (18) formula. The household was selected as the sampling unit, including all members in the study.

*n* = z^2^ *P* (1-P)/d^2^

Where, *n* = Sample size

*Z* = 1.96 (a confidence level of 95% was used)

*P* = average prevalence of malaria in the area

*D* = marginal error

The calculation was conducted by considering a 95% confidence interval for Z-statistics, which is conventionally 1.96% and 5% marginal error. The sample size was calculated using unpublished malaria prevalence data for the Humbo district report of 23.3% in 2016/2017. The sample size was calculated for every village to maximize study participants, which increases representativeness.

Assuming a 5% margin of error (d), a total of 275 households were included in the sample. Additionally, 25 households (9% of the sample) were allocated for contingency and non-response compensation, bringing the total number of households in each village to 300.

### Malaria case detection and patient management

Blood film collection was conducted by pricking the finger with a disposable blood lancet and all members of the household were tested for malaria parasites. Treatment was free and followed national and WHO malaria treatment guidelines (19, 20). Thick and thin blood smears were collected on the same slide, numbered, fixed using 100% methanol, and stained with 3% Giemsa solution for 20 min. Blood film examination was conducted according to WHO protocol (21) by two laboratory technicians in the Health Center using a light microscope. If there was disagreement between the two laboratory technicians, a third reader conducted a confirmatory examination. Slides were ruled positive or negative if two laboratory technicians agreed.

### Data management and analysis

The collected questionnaire data was analyzed using descriptive statistical tools such as percentage, mean, standard deviation, and a Chi-square test was performed to observe the impact of IRS on the malaria prevalence in Fango-Gelchecha village. SPSS version 20.0 software was used for statistical tests with a *P*-value of 0.05 for the significant test.

## Results

### Characteristics of study participants

A total of 3,075 individuals participated in the study. 1,646 were men (53.5%) and 1,429 were women (46.5%). 1,583 (51.5%) participants were from Fango-Gelchecha village, and 1,492 (48.5%) were from Shochora-Abela village. Most participants (70%; 2,152/3,075) were aged 15 and above (Table 1).

**Table 1:** Characteristics of the study participants by age, sex and village, August-December 2017.

Participants age	Participants sex	Villages		Total
		Fango-Gelchecha (%)	Shochora-Abela (%)	
<5 year	Male	28 (1.8)	57 (3.8)	85 (2.7)
	Female	24 (1.5)	49 (3.3)	73 (2.4)
	Total	52 (3.3)	106 (7.2)	158 (5.4)
5–14 year	Male	200 (12.6)	207 (13.8)	407 (13.2)
	Female	171 (10.8)	187 (12.5)	358 (11.6)
	Total	371 (23.4)	394 (26.4)	765 (24.9)
>15 year	Male	638 (40.3)	516 (34.6)	1,154 (37.5)
	Female	522 (32.98)	476 (31.9)	998 (32.4)
	Total	1,160 (73.3)	992 (66.5)	2,152 (70)
Overall	Male	866 (54.7)	780 (52.3)	1,646 (53.5)
	Female	717 (43.3)	712 (47.7)	1,429 (46.5)
	Total	1,583 (51.5)	1,492 (48.5)	3,075

### Prevalence of malaria

Out of 3,075 blood films examined in the two villages, 59 cases tested positive for malaria. The overall prevalence of malaria was 1.9% (95% CI: 1.5–2.5) with 10.1% (16/158) (95% CI: 5.9–15.9) prevalence in children under five, 4.7% (36/765) (95% CI: 3.3–6.4) in children aged 5–14 years, and 0.3% (95% CI: 0.13–0.67) in individuals over 15 years.

Concerning the parasite species, the highest prevalence of *P. falciparum* was 6.3% (95% CI: 3.1–11.3) among children aged less than five years, and it was 3.5% (95% CI: 2.3–5.1) in children aged 5–14 years (Table 2).

**Table 2:** The prevalence of *P. falciparum* and *P. vivax* malaria by age in Fango-Gelchecha and Shochora-Abela villages after IRS application, November to December 2017.

Participant age	Village	Number of malaria cases			*Pf* prevalence (95% CI)
		*P. vivax*	*P. falciparum*	No. negative	
<5 year	Fango-Gelchecha	2	7	43	13.5 (5.6–25.8)
	Shochora-Abela	4	3	99	2.8 (0.6–8.0)
	Total	6	10	142	6.3 (3.1–11.3)
5–14 year	Fango-Gelchecha	6	22	343	5.9 (3.7–8.8)
	Shochora-Abela	3	5	386	1.3 (0.4–2.9)
	Total	9	27	729	3.5 (2.3–5.1)
>15 year	Fango-Gelcheha	0	4	1,156	0.3 (0.1–0.8)
	Shochora-Abela	3	0	989	0.3 (0.1- 0.7)
	Total	3	4	2,145	0.18 (0.05 -0.47)
Total	Fango-Gelchecha	8	33	1,542	2.1 (1.4–2.9)
	Shochora-Abela	10	8	1,474	0.5 (0.2–1.1)
	Total	18	41	3,016	1.3 (0.9–1.8)

The prevalence of *P. falciparum* varies significantly among age groups. Children under five (OR = 36.1; 95% CI: 11.5–134.3; *P* < 0.001) and 5–14 years old (OR = 19.6; 95% CI: 7.3- 65.8; *P *< 0.001) had significantly higher odds of infection than those above 15 years old. The odds of *P. falciparum* malaria infection are significantly higher in the Fango-Gelchecha village (X2 = 12.9; OR = 3.9; 95% CI: 1.8–9.5; *P *< 0.001) than in Shochora-Abela village (Table 3).

**Table 3:** The number of participants tested for *P. falciparum* malaria and association between the outcome and selected variables, November to December 2017.

Variable		No. tested	No. Pf	X2 test	OR (95% CI)	*P*-value
Age	<5 year	158	10	92	36.1 (11.5–134.3)	<0.001 <0.001 1
	5–14 year	765	27	59.7	19.6 (7.3–65.8	
	>15 year	2,152	4	21.6	7.6 (3.5–32.9)	
Sex	Male	1,646	31	0.042	1.2 (0.86–2.1)	0.88 1
	Female	1,429	28	0.023	1.0 (0.62–1.75)	
Village	Fango-Gelchecha	1,583	33	12.9	3.9 (1.8–9.5)	<0.001
	Shochora-Abela	1,492	8	7.3	2.8 (0.9–7.6)	1

No. Pf, Number of Plasmodium falciparum positive.

### Village and species specific prevalence

The prevalence of *P. falciparum* was 2.1% (33/1,583; 95% CI: 1.4–2.9) in Fango-Gelchecha village and 0.5% (8/1,492; 95% CI: 0.2–1.1) in Shochora-Abela village. *Plasmodium vivax* was present in 0.5% (8/1,583; 95%CI: 0.2–0.9) of people in Fango-Gelchecha village and in 0.67% (10/1,492; 95%CI: 0.3–0.9) in Shochora-Abela village. Overall, *P. falciparum* was the most common malaria parasite accounting for 69.5% (95% CI: 56.1–80.8), and *P. vivax* was responsible for 30.5% (19.2–43.8). In Fango-Gelchecha village, *P. falciparum* accounted for 80.5% (95% CI: 65.1–91.2) of the cases, and in Shochora-Abela village, it accounted for 44.4% (21.5–69.2).

### Malaria prevalence in Fango-Gelchecha village before and after IRS

Before the IRS application, 54 out of 1,736 blood films were positive for malaria (prevalence: 3.1%, 95% CI: 2.3–4.0). Among positive cases, *P. falciparum* accounted for 79.6% and *P. vivax* accounted for 20.4%. After the IRS application, 41 out of 1,583 blood films were positive for malaria (prevalence: 2.6%, 95% CI: 1.8–3.5). Among positive cases, *P. falciparum* accounted for 80.5% and *P. vivax* accounted for 19.5%. The IRS application did not have a significant impact on the prevalence of malaria or the proportion of parasite species.

### Bed nets and IRS interventions

During the study, 536 out of 600 households (89.3%) had at least one mosquito net while 10.6% (64/600) of the households did not have a bed net. 56.5% (303/600) of houses had at least one bed net placed over the sleeping area, while 47.8% had no one sleeping under a bed net. During the peak mosquito season, 42.7% (192) of households use bed nets, and 57.3% (344) use them all year. Bed net usage was lowest among children aged 5–14 (4.2%).

Out of the 600 dwellings inspected in August 2017, only 351 (58.5%) were sprayed with insecticide. In Fango-Gelchecha village, 170 out of 300 (56.6%) were sprayed, while in Shochora-Abela village, 181 out of 300 (60.2%) were sprayed.

## Discussion

In this study, children had a higher prevalence of *P. falciparum* malaria than adults over 15. The use of ITNs was deficient in the age group between 5 and 14 years old despite relatively high coverage. The IRS coverage was considerably low and had no significant impact on malaria prevalence after three to four months of application.

The overall prevalence of malaria was 1.9% after applying IRS for three to four months. Prior to IRS, malaria prevalence was 3.1%; 3–4 months after IRS, it decreased to 2.6%. However, the IRS coverage was below the recommended 80% to significantly impact malaria prevalence (22). Although ITN coverage was high, actual use in the study villages was deficient. This is more important than coverage. Malaria prevalence did not significantly change despite similar bed net coverage and use rates before and after the IRS. This is likely because bed nets protect the user while poor IRS coverage offers little to no protection to individuals in unsprayed dwellings in the neighborhood (22, 23). In addition to coverage, the quality of spray and type of wall can affect efficacy of IRS (24).

The study found that *P. falciparum* was the most prevalent parasite, accounting for 69.5%. This species has been dominant in Ethiopia since Italian malarialogists investigated malaria epidemiology in the country (25, 26). The Malaria Eradication Service conducted a study in 30 locations, including southwest Ethiopia, in 1962 as part of a global campaign to eradicate malaria (25). Of the three documented parasites, *P. falciparum* was most prevalent, accounting for an average of 60%, followed by *P. vivax* and *P. malariae* (25). According to the World Health Organization, *P. falciparum* predominates nationwide (1). Despite decades of malaria control efforts, *P. falciparum* remains the dominant parasite, with only a few locations where *P. vivax* is prevalent (3). This indicates active malaria transmission caused by mosquito bites despite the government's targeting of *P. falciparum*.

The prevalence of *P. falciparum* malaria varies by age group, with children having a higher infection rate than those over 15. The higher prevalence of malaria in children implies active local malaria transmission, possibly due to insufficient coverage and use of IRS and ITN. Studies show that children who do not use ITNs have a higher prevalence of malaria (27, 28). Moreover, the application of IRS did not significantly reduce malaria prevalence, possibly due to inadequate population coverage. Several studies have shown that the IRS needs to cover more than 80% of the population to succeed (22, 23).

This study has both strengths and limitations. Despite being cross-sectional, its large sample size, active case-finding approach via house-to-house visits, and inclusion of all age groups are believed to enhance its representativeness. Analyzing the results of the regular malaria control programme IRS implementation may provide a better understanding of the situation. Since malaria microscopists' capacity is limited, and asymptomatic infections have low parasite density, many cases may be missed through microscopy. Additionally, the impact of IRS was analyzed in only one village, so a more comprehensive analysis could be conducted if it were performed in both villages.

## Conclusions

The study found that *P. falciparum* was the most common malaria parasite in the surveyed villages, affecting children more often than adults. Therefore, children require special attention for protection. Although a regular malaria control program has been put in place through the IRS, there has been no notable decrease in malaria prevalence even after three to four months. To effectively combat malaria, it is essential to expand the coverage of the IRS and conduct a long-term study to evaluate its effectiveness.

## Data Availability

The original contributions presented in the study are included in the article/Supplementary Materials, further inquiries can be directed to the corresponding author.

## References

[1] WHO. World malaria report: 20 years of global progress and challenges. Geneva: World Health Organization (2020)

[2] WHO. World malaria report 2016. Geneva: World Health Organization (2016)

[3] Taffese HS Hemming-Schroeder E Koepfli C Tesfaye G Lee MC Kazura J Malaria epidemiology and interventions in Ethiopia from 2001 to 2016. Infect Dis Poverty (2018) 7: 103 10.1186/s40249-018-0487-3 30392470 PMC6217769

[4] Esayas E Woyessa A Massebo F. Malaria infection clustered into small residential areas in lowlands of southern Ethiopia. Parasite Epidemiol Control (2020) 10: e00149. 10.1016/j.parepi.2020.e00149 32368628 PMC7190761

[5] Massebo F Balkew M Gebre-Michael T Lindtjorn B. Entomologic inoculation rates of *Anopheles arabiensis* in southwestern Ethiopia. Am J Trop Med Hyg (2013) 89: 466–73. 10.4269/ajtmh.12-0745 23878184 PMC3771283

[6] Animut A Balkew M Gebre-Michael T Lindtjørn B. Blood meal sources and entomological inoculation rates of anophelines along a highland altitudinal transect in south-central Ethiopia. Malar J (2013)12: 79. 10.1186/1475-2875-12-79 23433348 PMC3626914

[7] Abraham M Massebo F Lindtjørn B. High entomological inoculation rate of malaria vectors in area of high coverage of interventions in southwest Ethiopia: implication for residual malaria transmission. Parasite Epidemiol Control (2017) 2: 61–9. 10.1016/j.parepi.2017.04.003 29774282 PMC5952686

[8] Bhatt S Weiss DJ Cameron E Bisanzio D Mappin B Dalrymple U The effect of malaria control on *Plasmodium falciparum* in Africa between 2000 and 2015. Nature (2015) 526: 207–11. 10.1038/nature15535 26375008 PMC4820050

[9] Killeen GF. Characterizing, controlling and eliminating residual malaria transmission. Malar J (2014) 13:330 10.1186/1475-2875-13-330 25149656 PMC4159526

[10] Carnevale P Manguin S. Review of issues on residual malaria transmission. J Infect Dis (2021) 223: 61–80. 10.1093/infdis/jiab084 PMC8079138 33906221

[11] Guyant P Corbel V Guérin PJ Lautissier A Nosten F. Past and new challenges for malaria control and elimination: the role of operational research for innovation in designing interventions. Malar J (2015) 14:279 10.1186/s12936-015-0802-4 26185098 PMC4504133

[12] Alonso PL Tanner M. Public health challenges and prospects for malaria control and elimination. Nat Med (2013)19: 150–5. 10.1038/nm.3077 23389615

[13] Loha E Lindtjørn B. Model variations in predicting incidence of plasmodium falciparum malaria using 1998-2007 morbidity and meteorological data from south Ethiopia. Malar J (2010) 9:166 10.1186/1475-2875-9-166 20553590 PMC2898788

[14] Legesse D Haji Y Abreha S. Trend analysis of malaria occurrence in Wolaita Zone, Southern Ethiopia: retrospective cross-sectional study. Malar Res Treat (2015) 2015:123682. 10.1155/2015/123682 26770866 PMC4685134

[15] Snow RW Sartorius B Kyalo D Maina J Amratia P Mundia CW The prevalence of *Plasmodium falciparum* in sub-saharan Africa since 1900. Nature (2017) 550:515–8. 10.1038/nature24059 29019978 PMC5660624

[16] Price RN Commons RJ Battle KE Thriemer K Mendis K. *Plasmodium vivax* in the era of the shrinking *P. falciparum* map. Trends Parasitol (2020) 36:560–70. 10.1016/j.pt.2020.03.009 32407682 PMC7297627

[17] Cohee LM Opondo C Clarke SE Halliday KE Cano J Shipper AG Preventive malaria treatment among school-aged children in sub-saharan Africa: a systematic review and meta-analyses. Lancet Glob Heal (2020) 8:e1499–511 10.1016/S2214-109X(20)30325-9 PMC7721819 33222799

[18] Bartlett JE Kotrlik JW Higgins CC. Organizational research: determining appropriate sample size in survey research. Inf Technol Learn Perform J (2001) 19:43–50.

[19] FMOH. National Malaria Guidelines, Fourth edition. Addis Ababa: Ministry of Health (2017)

[20] WHO. WHO Guidelines For Malaria. Geneva: World Health Organization (2022)

[21] Garcia LS. Malaria. Clin Lab Med (2010) 30: 93–129. 10.1016/j.cll.2009.10.001 20513543

[22] Zhou Y Zhang WX Tembo E Xie MZ Zhang SS Wang XR Effectiveness of indoor residual spraying on malaria control: a systematic review and meta - analysis. Infect Dis Poverty (2022)11:83 10.1186/s40249-022-01005-8 35870946 PMC9308352

[23] Rehman AM Coleman M Schwabe C Baltazar G Matias A Gomes R How much does malaria vector control quality matter: the epidemiological impact of holed nets and inadequate indoor residual spraying. PLoS One (2011) 6: e19205 10.1371/journal.pone.0019205 21559436 PMC3084796

[24] Desalegn Z Wegayehu T Massebo F. Wall - type and indoor residual spraying application quality affect the residual efficacy of indoor residual spray against wild malaria vector in southwest Ethiopia. Malar J (2018) 17:300 10.1186/s12936-018-2458-3 30126433 PMC6102920

[25] Schaller KF. *Medizinische Landerkunde Athiopien-Ethiopia*. Vol 3 of Geomedical Monograph Series (1972) 159 pp.

[26] National Malaria Control Team, Ethiopian Public Health Institute, World Health Organization, Addis Ababa University and the INFORM Project. An epidemiological profile of malaria in Ethiopia. A report prepared for the Federal Ministry of Health, Ethiopia, the Roll Back Malaria Partnership and the Department for International Development, UK (2013)

[27] Korenromp EL Miller J Cibulskis RE Cham MK Alnwick D Dye C. Monitoring mosquito net coverage for malaria control in Africa: possession vs. Use by children under 5 years. Trop Med Int Heal (2003) 8:693–703. 10.1046/j.1365-3156.2003.01084.x 12869090

[28] Biset G Tadess AW Tegegne KD Tilahun L Atnafu N. Malaria among under-five children in Ethiopia: a systematic review and meta-analysis. Malar J (2022) 21:338 10.1186/s12936-022-04370-9 36384533 PMC9667600

